# Semi-batch synthesis of colloidal spheres with fluorinated cores and varying grafts of poly(ethylene glycol)

**DOI:** 10.1007/s00396-017-4172-z

**Published:** 2017-08-15

**Authors:** G. Kristin Jonsson, Jeanette Ulama, Malin Johansson, Malin Zackrisson Oskolkova, Johan Bergenholtz

**Affiliations:** 1 Department of Chemistry and Molecular Biology, University of Gothenburg, 41296 Göteborg, Sweden; 2 Division of Physical Chemistry, Center of Chemistry and Chemical Engineering, Lund University, 22100 Lund, Sweden

**Keywords:** PEG, Fluorinated colloids, Emulsion polymerization, Monodisperse spheres, SAXS, Crosslinking

## Abstract

Fluorinated spheres with grafted poly(ethylene glycol) (PEG) have been synthesized using a semi-batch emulsion polymerization in which the initiator is fed slowly to the reaction. In this way, PEG-grafted colloidal spheres can be fabricated with varying PEG chain length, different cores and varying degrees of crosslinking. The resulting batches have been characterized using disc centrifuge photosedimentometry and small-angle X-ray scattering. The size distribution is shown to be a sensitive function of the molar ratio of the reactive PEG macromonomer to fluorinated monomer, and with some optimization latices of very low polydispersity can be obtained with this simple synthesis method. For short PEG grafts too high a molar ratio results in a build up of smaller size particles and a broadening of the size distribution, whereas for longer grafts the mean particle size increases with decreasing molar ratio.

## Introduction

Colloidal suspensions of fluorinated particles stand to find uses in coatings applications [1–6], as optical devices [7, 8], low-refractive-index particles and media for microscopy [9–12], and as model colloidal systems for a wide range of fundamental studies [13–20]. Such fluorinated particles are usually synthesized via conventional emulsion polymerization [21–23] or mini- [24–26] and microemulsion [27] polymerization. Generally, these methods employ emulsifiers/stabilizers, which, although they impart improved stability to suspensions, are undesired in biomedical applications. In this context, adding grafted poly(ethylene glycol) surface layers for minimizing protein adsorption and for prolonging in vivo circulation times is a strategy that is often used and may open up for applications in the biomedical and pharmaceutical fields [28, 29]. Surface-grafting of PEG on fluorinated particles has been achieved using various polymerization schemes [19, 30]. A particularly simple, semi-batch emulsion polymerization was recently suggested for producing nearly monodisperse spherical particles with a well-defined surface layer of PEG [31] (referred to henceforth as I). The PEG graft was inferred to be of high grafting density as the brush thickness was several times the bulk radius of gyration of unattached PEG molecules of the same molecular weight. In line with this observation, the systems exhibited an extreme stability against salt-induced aggregation. However, studies of this synthesis method have so far been restricted to a single PEG molecular weight.

In conventional batch emulsion polymerization, the monomer, stabilizers, and initiator, are all present at the outset, whereas in semi-batch or semi-continuous emulsion polymerization one or more components are added incrementally or continuously to the reacting system. Thus, these latter methods open up a large parameter space for tailoring the end product, such as the morphology [32, 33] and size distribution [34, 35] of polymer particles. In studies of unseeded semi-batch syntheses, it is by far most common to work with various monomer feeding schemes. However, in I, in an attempt to reduce PEG-PEG termination in standard batch emulsion polymerization [22, 36], the initiator was instead added throughout the first several hours of the reaction. This was key to obtaining nearly monodisperse PEG-grafted spheres containing fluorinated polymer. Separate addition of initiator has been used in emulsion homo-polymerization to yield particles with a narrow size distribution [37] and controlling the initiator feed rate has been predicted to produce narrow molecular weight distributions in free radical solution polymerization [38].

In this work, it is shown that the semi-batch emulsion polymerization approach readily accommodates a range of PEG molecular weights, as well as internal crosslinking, control of particle size, and use of other monomers, while maintaining a low degree of polydispersity.

## Experimental

### Materials

The fluorinated monomer 2, 2, 3, 3, 4, 4, 4-heptafluorobutyl methacrylate (HFBMA, 97%), stabilized by hydroquinone, was obtained from Alfa Aesar. The inhibitor was removed prior to use by passing the monomer through a column packed with material for inhibitor removal (CAS 9003-70-7, Alfa Aesar). Styrene (St, 99.5%), methoxy poly(ethylene glycol) acrylate (mPEGA5000) with an average molecular weight (MW) of 5000 g/mol and ethylene glycol dimethacrylate (EGDMA) (all three supplied by Sigma Aldrich) were also treated in the same manner to remove any polymerization inhibitor. Methoxy poly(ethylene glycol) acrylate (mPEGA480) with an average MW of 480 g/mol (Sigma Aldrich) was stabilized by butylated hydroxytoluene (BHT) and methyl ether hydroquinone (MEHQ). It was purified using a similar packed column with a mixture of inhibitor removal (CAS 9003-70-7, Alfa Aesar) and Al_2_O_3_ (Fluka, purum p.a.). The initiator, potassium persulfate (KPS, Sigma Aldrich), was recrystallized in water once prior to use. Dodecane (99%, Sigma Aldrich) was used as received. NaCl (99.5%) and Na_2_CO_3_ (99.9%) (both supplied by Merck), NaN_3_ (analytical reagent grade, Fisher Scientific) were all used as received. Colloidal polyvinyl chloride (PVC) particles (CPS instruments Inc.) and sucrose (Fluka) were also used as received, as well as tetrahydrofuran (THF, 99.9%, VWR Chemicals). For purification of dispersions dialysis tubes with a cut-off MW of 12–14 kDa (MAKAB) or 300 kDa (Spectra/por) were used depending on the molecular weight of the PEG macromonomer.

### Methods

Dynamic light scattering (DLS) measurements were performed using a Malvern Zetasizer Nano ZS, equipped with a He-Ne laser with a wavelength of 633 nm and a detector positioned at a scattering angle of 173°. The hydrodynamic radius was extracted from a second-order cumulant analysis at 25 °C.

An instrument (model DC18000, CPS Instruments) for disc centrifuge photosedimentometry (DCP) was used to determine particle size distributions. The instrument employs a detection wavelength of 405 nm and records the apparent absorption of particles sedimenting in a density gradient generated by sucrose (1–8 or 8–22 wt %) solutions, sealed with dodecane to prevent evaporation. Colloidal PVC particles with a diameter of 239 nm were used to quantify the density gradient and the disc rotation speed was set to 15000 or 18000 rpm depending on the particle size. The polydispersities (cf. Table 2) were calculated as the standard deviation normalized by the mean.

Small-angle X-ray scattering (SAXS) measurements were carried out at the ID02 beamline at the European Synchrotron Radiation Facility (ESRF) in Grenoble, France. All scattering measurements employed a wavelength *λ* of 0.995 Å and sample-to-detector distances of 10 and 20 m leading to a total scattering vector (*q*) range of 0.0035 ≤ *q* ≤ 0.76 nm^−1^, where *q* = 4*π* sin(*𝜗*/2)/*λ* in terms of the scattering angle *𝜗*. A flow-through quartz capillary with an inner diameter of 1.6 mm was used for all dispersions and solvent backgrounds. Exposure times varied between 0.1 and 1 s depending on the particle concentration. Typically, after a preliminary check for sample beam damage, 10 consecutive spectra were recorded. These were individually averaged radially and examined for beam damage, before they were included in the final averaged scattering spectrum. Solvent scattering, measured in the same capillary, was subtracted as background and a measurement of water was used to bring the scattering curves onto an absolute scale.

Additional SAXS measurements were made at the Division of Physical Chemistry, Lund University, Sweden, on an automated SAXS pinhole system (Ganesha, JJ X-Ray A/S, Denmark). The instrument is equipped with a microfocus sealed tube source with shaped multilayer optics and a two-dimensional single-photon counting solid-state Pilatus detector (Dectris Ltd, Switzerland). Data were recorded using either a 2- or 3-pinhole collimation configuration, employing scatterless apertures. The highest resolution, 3-pinhole configuration, referred to as configuration 4 in [39], was employed for the fluorinated particles. The 2-pinhole configuration, referred to as configuration 26 in [39], was used for the grafted polystyrene particles, which required significantly longer measurement times due to a much lower scattering contrast. In both cases, the sample-to-detector distance was 1491.7 mm. Experimental data were processed and radially averaged using the SAXSGUI software as a function of *q* with, in this case, *λ* = 0.154 nm (Cu K *α* line). The scattering from the aqueous solvent in the same capillaries was measured as background and was subtracted to yield the excess scattering. These scattering data were not converted to an absolute intensity scale.

Cryo-electron microscopy was carried out at the Department of Biosciences and Nutrition at Karolinska Institutet, Huddinge, Sweden. Samples of 3.5 *μ* L were applied to glow-discharged holey carbon-coated copper grids in a FEI Vitrobot, using a controlled environment of 22 °C and a relative humidity close to 100%. After blotting excess liquid away, samples were plunged into liquid ethane. The vitrified samples were transferred in a Gatan 626 cryo-transfer holder into a JEOL JEM-2100f microscope, operated at 200 kV acceleration voltage. Images were recorded with a TVIPS TemCam-F415 4k × 4k CCD-camera (Tietz Video and Image Processing Systems GmbH, Gauting, Germany) under low-dose conditions at 1 *μ* m defocus.

### Synthesis

The synthesis procedure employed has been described in some detail elsewhere [31] and will only be briefly covered here. The stirring rate during the polymerization was maintained at 150 rpm for all batches and the sodium bisulfite used in some of the previous work was left out. As before, after charging the 250-mL flask with water, HFBMA monomer, and PEG macromonomer, and emulsifying, 10 mL of a KPS solution was added dropwise during ∼ 3 h. For the crosslinked particles, the EGDMA crosslinker was added after the PEG and HFBMA additions. The compositions of the different synthesized batches are listed in Table 1. All dispersions were filtered and dialyzed against MilliQ water, after which 10 mM electrolyte (7 mM NaCl and 3 mM NaN_3_) was added. Sodium azide is added to prevent bacterial growth and sodium chloride is added to screen any residual surface charges stemming from the initiator. Yields (cf. Table 2) were calculated in percent of the dry weight of the dialyzed and filtered dispersions divided by the total mass.

**Table 1:** Synthesized batches of PEG-grafted particle dispersions in terms of mPEGA/monomer molar ratio *X*, type of monomer, PEG molecular weight, EGDMA/HFBMA molar ratio *Y*, and amounts of initiator and monomer

Batch	*X*	Core	PEG MW	*Y*	K_2_S_2_O_8_	Monomer
			g/mol		mg	mL
LP5	0.05	HFBMA	480	–	11.7	1
LP10	0.10	HFBMA	480	–	11.7	1
LP20	0.20	HFBMA	480	–	11.7	1
LL10	0.010	HFBMA	5000	–	11.7	1
LL15	0.015	HFBMA	5000	–	11.7	1
LL25	0.025	HFBMA	5000	–	11.7	1
LL45	0.045	HFBMA	5000	–	11.7	1
L5sm	0.05	HFBMA	2000	–	11.7	0.5
PSSB	0.038	Styrene	2000	–	56	0.7
PSB	0.038	Styrene	2000	–	56	0.7
LL25-2	0.025	HFBMA	5000	0.02	11.7	1
LL25-5	0.025	HFBMA	5000	0.05	11.7	1
LL25-8	0.025	HFBMA	5000	0.08	11.7	1

**Table 2:** Resulting yield, hydrodynamic radius R_H_ (from DLS), mean core radius $\bar {{\mathrm {R}}}_{\text {core}}$ (from SAXS), and polydispersity (from DCP) for the synthesized batches

Batch	Yield	R_H_	$\bar {{\mathrm {R}}}_{\text {core}}$	$\sigma /{\bar {\mathrm {R}}}$
	%	nm	nm	
LP5	13	122	120	0.03
LP10	22	123	–	0.06
LP20	62	105	–	0.10
LL10	22	119	111	0.04
LL15	32	90	–	0.09
LL25	34	64	57	0.06
LL45	38	37	27	0.13
L5sm	21	59	–	0.07
PSSB	44	48	–	–
PSB	70	48	–	–
LL25-2	21	55	–	–
LL25-5	20	63	–	–
LL25-8	21	79	–	–

## Scattering model

For dilute systems, the scattering intensity *I*(*q*) is given by the number density of particles *n* times the so-called form factor

1 $$ I(q) = n\left\langle F(q)^{2}\right\rangle = n{\int}_{0}^{\infty} F(q)^{2}f(R)dR  $$

where the form amplitude *F*(*q*) in this case is taken as that for a homogeneous sphere with radius *R* and excess electron density Δ*ρ*,

$$\begin{array}{@{}rcl@{}} F(q)=4\pi {\Delta} \rho (\sin qR-qR\cos qR )/q^{3}. \end{array} $$

We model the size distribution as a sum of two Gaussian distributions $f(R)=x_{1}f_{1}(R; \bar {R}_{1},\sigma _{1})+x_{2}f_{2}(R; \bar {R}_{2},\sigma _{2})$, centered on the average radii $\bar {R}_{1}$ and $\bar {R}_{2}$, respectively, with standard deviations *σ*_1_ and *σ*_2_. With these Gaussian distributions, each normalized to unity, the mole fractions of the two populations satisfy *x*_1_ + *x*_2_ = 1. For sufficiently narrow size distributions, the lower integration limit in Eq. 1 can be extended to −*∞* [40] and the integral can be completed analytically to yield

2 $$\begin{array}{@{}rcl@{}} \left\langle F(q)^{2}\right\rangle &\,=\,& \left( \frac{4\pi {\Delta} \rho }{q^{3}}\right)^{2} \!\left( x_{1}G(q\bar{R}_{1},q\sigma_{1})\,+\,x_{2}G(q\bar{R}_{2},q\sigma_{2})\right) \\ & & \end{array} $$

where

3 $$\begin{array}{@{}rcl@{}} G(x,y) &\,=\,& \frac{1}{2}\left\{ 1\,+\,x^{2}+y^{2}-2x(1\,+\,2y^{2})e^{-2y^{2}}\sin(2x) \right. \\ & & \left. \!+(x^{2}-3y^{2}-4y^{4}-\!1)e^{-2y^{2}}\cos (2x) \right\} \end{array} $$

with $x=q\bar {R}$ and *y* = *q* *σ*.

## Results and discussion

In I, slow feeding of initiator solution was employed to the reaction vessel containing the fluorinated monomer emulsion with dissolved PEG macromonomer to produce spherical PEG-grafted particles. The polydispersity could be reduced to low values by keeping a relatively low molar ratio, *X*, of PEG macromonomer to fluorinated monomer. The resulting grafting density was inferred, from the extreme stability against addition of salt and the low electrophoretic mobilities, to be high. In addition, the PEG-graft thickness measured to several times the bulk radius of gyration of free PEG of the same molar mass, suggesting an extended, brush-like conformation of the PEG graft. Parameters such as the stirring rate and the presence of sodium bisulfite were found to play only minor roles. However, the entire study was restricted to PEG-grafts with a molecular weight of 2000 g/mol. Thus, it is of interest to examine how flexible this simple semi-batch scheme is. To this end, we employ the same procedure as in I but with varying molecular weight of the PEG macromonomer. In addition, three syntheses are made with a crosslinker added in order to crosslink the particles covalently. Finally, we apply the semi-batch procedure to synthesize PEG-grafted polystyrene particles and compare with similar particles synthesized via a standard batch procedure. In Table 1, the compositions and batch labels are given for the various syntheses.

### Preparation of particles with short and long PEG grafts

**Fig. 1:**
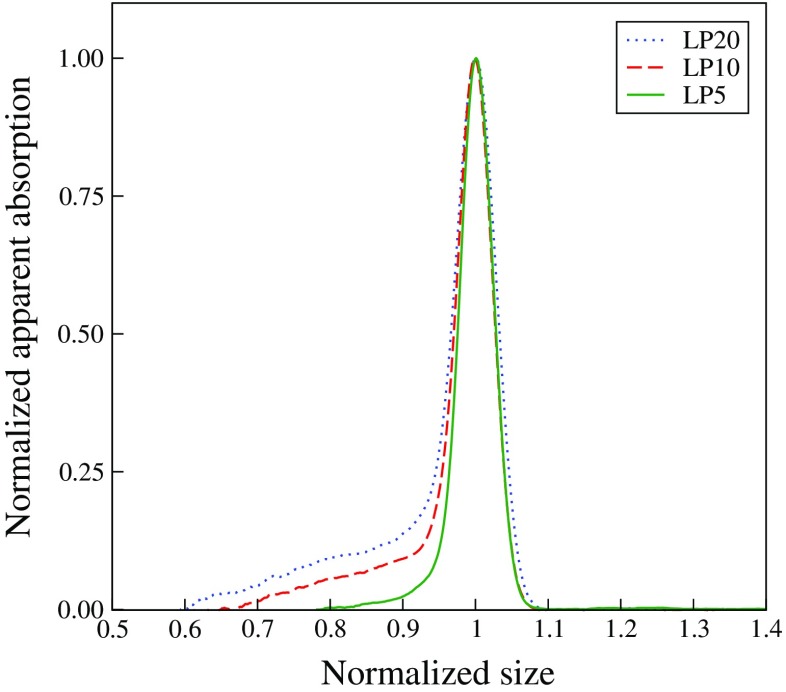
Size distribution of pHFBMA particles with short PEG grafts (480 g/mol) as determined by DCP, as labeled. For clarity, the apparent absorption and sizes have been normalized to give unity at the main peak

For short PEG480 grafts, similar molar ratios were employed as before in I when PEG2000 was used. As seen in Table 2, for the lower PEG macromonomer/fluorinated monomer molar ratios particles with radii around 120 nm are produced. When the molar ratio is increased, the particle size becomes somewhat smaller and the polydispersity increases, which was also observed for particles grafted with PEG2000 in I. The size distribution of the particles was determined by DCP, which records an apparent absorption mostly due to scattering. The results are shown in normalized form in Fig. 1. The normalization reveals that regardless of the molar ratio, *X*, a narrow main peak is obtained in the size distribution. However, increasing the molar ratio leads to a gradual build up of smaller sized particles that has the effect of increasing the overall polydispersity as reported in Table 2.

**Fig. 2:**
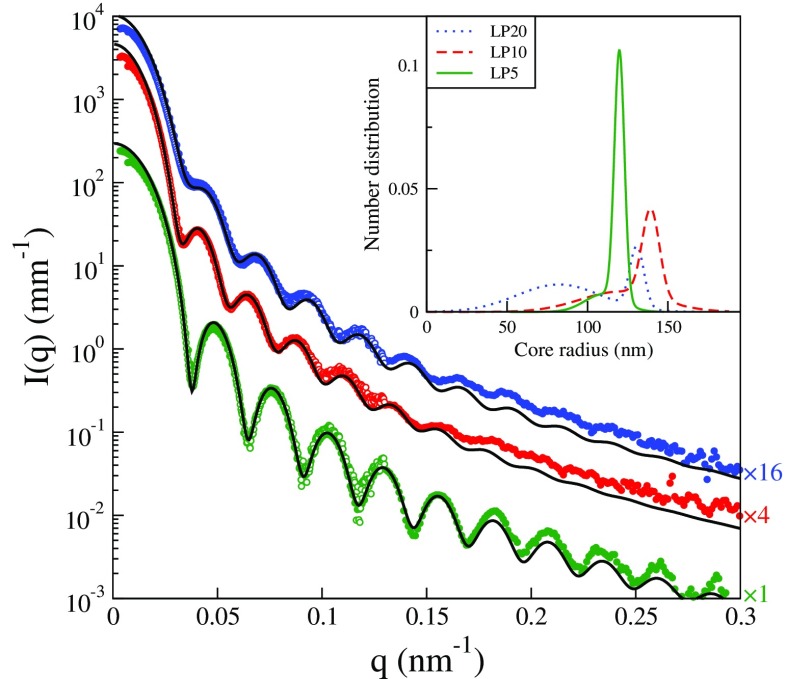
Scattering intensity as a function of *q* for dilute dispersions of, from *top-to-bottom*, LP20, LP10, and LP5. Some *scattering curves* have been shifted vertically for clarity using the factors given on the *right in the figure*. The *open symbols* are data recorded with a sample-to-detector distance of 20 m, whereas *filled symbols* are data recorded at 10 m. The *solid lines* are fitted form factors of homogeneous spheres with size distributions shown in the *inset*

Further characterization has been done using SAXS on dilute dispersions of the particles. Under these conditions the so-called form factor is determined and it is possible to observe the effect of the broadening of the size distribution with increasing molar ratio. As seen in Fig. 2, the narrow main peak of the LP5 particles results in a large number of minima in the form factor as a function of scattering vector. With increasing molar ratio, going to LP10 and then to LP20, these minima become more shallow. This holds particularly for the first minimum, at *q* ∼ 0.035 nm^−1^, which is replaced by a hump.

In analyzing the SAXS data for particles with short PEG grafts, it becomes clear that the high fluorine content of the pHFBMA cores completely dominates the scattering signal, as also noted in I for PEG2000, such that the data can be described by the form factor for homogeneous spheres. However, quantitative agreement between scattering model and data requires that the model allow for non-symmetric size distributions of the sort observed in the DCP measurements in Fig. 1. For this reason, we have employed a number-based size distribution that is a superposition of two Gaussian distributions. The scattering data can in this way be quantitatively modeled as shown in Fig. 2. The size distributions corresponding to the form factor fits are shown in the inset to Fig. 2. In agreement with the DCP measurements in Fig. 1, these size distributions exhibit a qualitatively similar relative increase in smaller size particles with increasing molar ratio. As noted by Pontoni et al. [41], the most common unimodal size distributions cannot be used to describe a preferential smearing of the first form factor minimum while leaving relatively well-defined minima at higher *q*. To reproduce this effect they used a size distribution skewed to give a tail of larger particles. As noted here, a tail toward smaller size particles can produce a similar effect. However, for low molar ratios the production of smaller size particles is essentially completely suppressed and a very low polydispersity, around 3*%*, results.

**Fig. 3:**
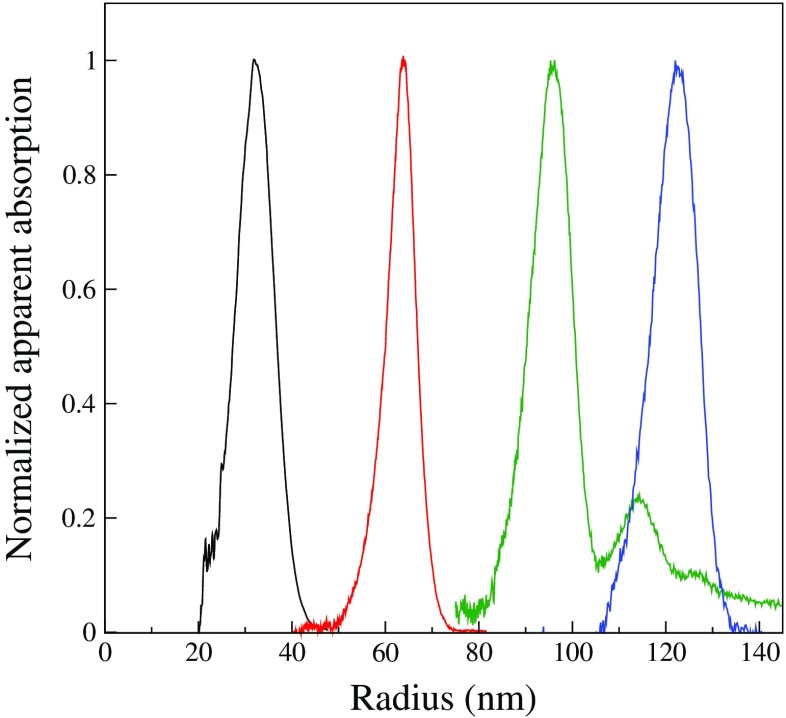
Size distributions, determined by DCP, of pHFBMA particles with longer PEG5000 grafts synthesized with different molar ratios of PEG macromonomer to fluorinated monomer, form *left-to-right*: LL45, LL25, LL15, and LL10. For clarity, the apparent absorption recorded by the instrument has been normalized to a peak value of unity

Increasing the PEG graft chain length, by using mPEGA5000 as a macromonomer in the synthesis, requires lowering the molar ratio, *X*, but no other adjustments of the synthesis procedure were needed. A series of four dispersions, LL10-LL45 in Table 1, were synthesized using molar ratios in the range 0.01 ≤ *X* ≤ 0.045. The size distributions of the four dispersions, as determined by DCP, are shown in Fig. 3. These show a strong size dependence on the molar ratio with the particle size increasing with decreasing molar ratio. In contrast to the particles with short PEG grafts, no tails toward smaller size particles can be seen in the size distributions. Figure 3 also shows that the absolute width of the size distributions from DCP is relatively constant, though LL25 is somewhat more narrow. However, the polydispersity, usually defined as the standard deviation divided by the mean, increases with increasing *X* mainly because the mean particle size decreases. Batch LL15 was found to contain a secondary peak in the size distribution and it was excluded from further analysis.

**Fig. 4:**
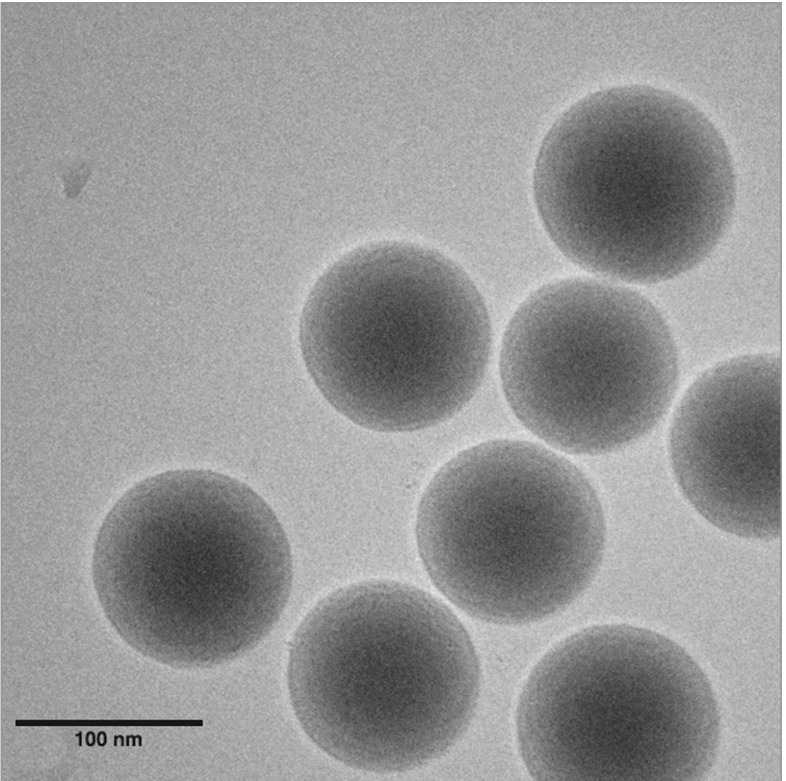
Cryo-EM image of the LL25 dispersion

In Fig. 4, a cryo-EM image of the LL25 dispersion is shown. As in I, the synthesized particles were found to be spherical and one can observe in Fig. 4 that the particles do not come into intimate contact with one another, which is expected for sterically stabilized particles. A mean radius and a polydispersity (standard deviation divided by mean) was determined as 51.5 nm and 0.06 using a limited set of 40 imaged particles.

**Fig. 5:**
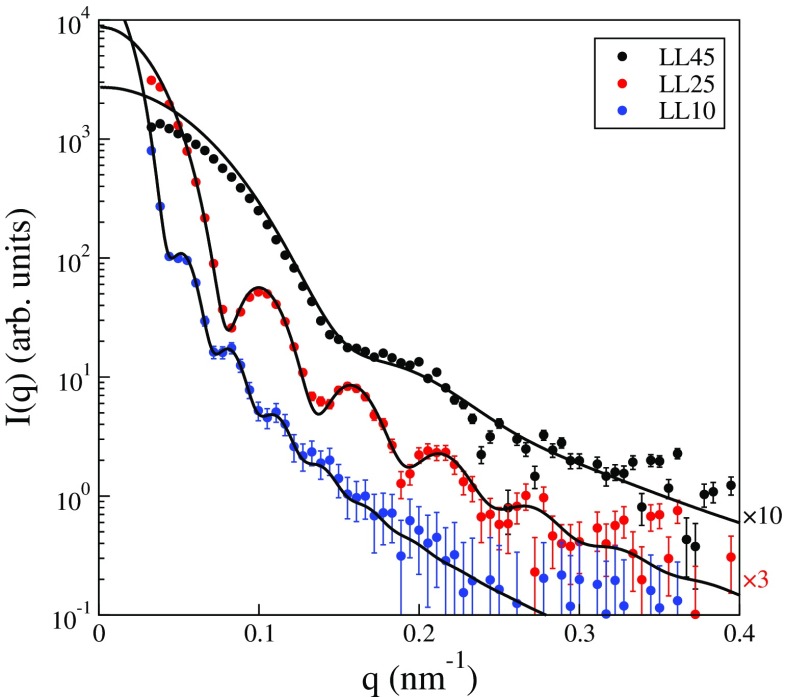
Scattering intensity recorded as a function of *q* using a 3-pinhole configuration for dilute dispersions of, from *top-to-bottom*, LL45, LL25, and LL10. Some *scattering curves* have been shifted vertically for clarity using the factors given on the *right in the figure*. The *solid lines* are fitted form factors of unimodally distributed homogeneous spheres

These particles with longer PEG grafts were also examined using SAXS, though this time with an in-house laboratory instrument rather than at a synchrotron facility. As a consequence, instrumental smearing related to the finite beam width and divergence affect the scattering from dispersions of larger size particles (LL25 and LL10 in Fig. 5). However, this was taken into account in the analysis by smearing the results of the form factor model in Eq. 2 using the experimentally measured beam profile [39]. As seen in Fig. 5, the difference in the particle size as a function of molar ratio is evident also in the scattering from the dispersions. The form factor oscillations shift along the *q*-axis in familiar fashion as the particle size changes. The limitations in the instrumental resolution preclude drawing direct conclusions as to the polydispersity from the number and depth of the form factor minima and quantitative model fitting is necessary. Here, guided by the DCP measurements in Fig. 3, a unimodal size distribution is employed to model the scattering data. This allows for nearly perfect fits to the scattering from the dispersions with the two larger particle sizes using only a mean radius and a polydispersity as fitting parameters. The same model does not work nearly as well for the scattering from the LL45 dispersion, which contains the smallest particles. The mismatch between data and model at lower *q* may suggest structure factor effects, but it is more likely that the scattering from the PEG graft begins to contribute when the core size becomes smaller. This was neglected in the model.

Due to the strongly scattering pHFBMA cores, the PEG-grafts did not contribute to the scattering intensities for the shorter PEG grafts. Therefore, the analysis of the SAXS data by form factor fitting yields the radius of the particle cores. The average values of these core radii, $\bar {{\mathrm {R}}}_{\text {core}}$, have been reported in Table 2 for the systems which exhibited a predominantly unimodal size distribution. However, when it comes to the particles with the longer, PEG5000 graft, we note that the size distribution from analysis of cryo-EM images of LL25 results in a mean radius of 51.5 nm, whereas the SAXS modeling requires a significantly larger radius of 57 nm. The polydispersities obtained from the two analyses are in excellent agreement. In I, a similar SAXS and cryo-EM analysis resulted in close agreement as to the core radius, assuming the PEG graft does not contribute to the scattering intensity. The reason for the discrepancy found here is likely a scattering contribution from these longer PEG grafts. It should also be noted that the LL25 particles are smaller than the LP particles and the relative contribution to the scattering from the PEG graft is expected to be greater.

The stability against salt-induced aggregation was tested using aqueous Na_2_CO_3_ solutions. Most of the synthesized dispersions accommodate 0.5 M Na_2_CO_3_ without showing signs of aggregation under dilute particle concentrations. However, the two batches, LL10 and LL15, synthesized at low molar ratios, aggregate at significantly lower Na_2_CO_3_ concentrations, suggesting that the grafting density at these lower molar ratios is not as high. The stability behavior of the dispersions as a function of PEG-graft chain length will be reported in a separate publication.

In I, pHFBMA particles were synthesized with grafted PEG2000. For a molar ratio of *X* = 0.05 the semi-batch procedure resulted in particles of roughly 100 nm radius with a narrow size distribution. The particle size was found to vary strongly with molar ratio for particles with PEG5000 grafts, whereas such a systematic variation was not evident for the particles with the PEG480 grafts. However, it is important to be able to control the particle size using other parameters than the molar ratio because it has such a strong influence on the stability of the dispersions. The particle size is generally affected by numerous variables in batch emulsion polymerization, one of which is the monomer concentration [21, 22, 42]. Here, the semi-batch method was employed keeping all variables the same as for the L5 batch with PEG2000 in I, except for reducing the concentration of the fluorinated monomer by a factor of two. This synthesis resulted in batch L5sm as reported on in Tables 1 and 2. The reduction in monomer concentration gave particles with a much smaller hydrodynamic radius of 59 nm and the same stability limit against added Na_2_CO_3_ as that reported in I for L5. The polydispersity, reported in Table 2, was 7% but some broadening due to diffusion during the disc centrifuge measurement might be expected for this smaller particle size. The reduced size of the particles of L5sm suggests that the particle size can be controlled simply by adjusting the HFBMA concentration.

### Crosslinked particles

PEG is a tremendously versatile polymer in that, e.g., it is not only soluble in water but also in a variety of other liquids. However, care needs to be taken when dispersing PEG-grafted particles in non-aqueous media because particle cores may swell. The swelling can be kept within bounds by crosslinking the polymer in the particle cores. Crosslinking of fluorinated particles has been previously reported by Alteheld et al. [8] and Pan et al. [7] using EGDMA crosslinker, though without verifying that the latices actually became crosslinked.

**Fig. 6:**
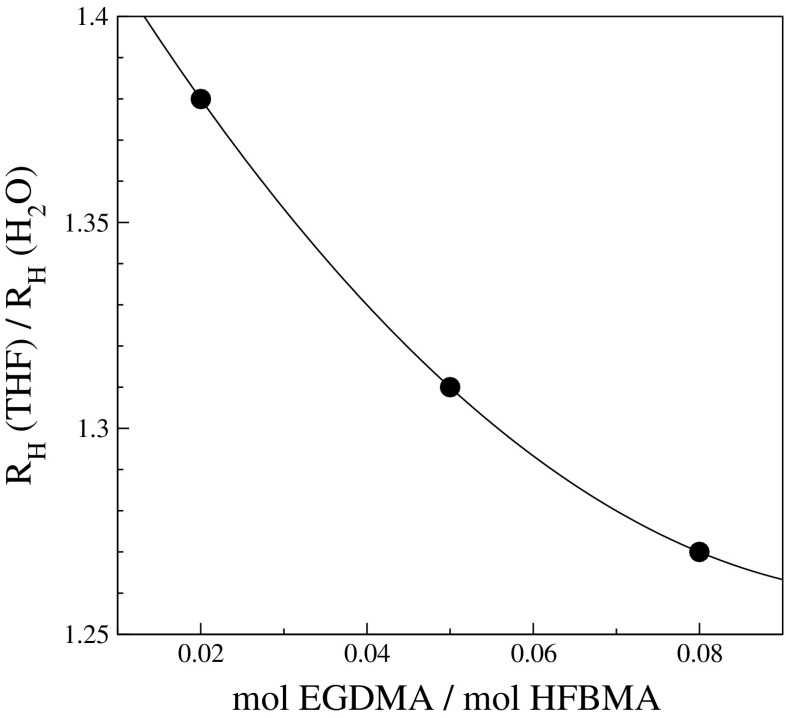
The hydrodynamic radius in THF divided by the hydrodynamic radius in water (R_H_(THF)/R_H_(H_2_O)) as a function of EGDMA/HFBMA molar ratio. The *line* is shown as a guide to the eye. The data point for LL25, corresponding to a molar ratio of 0, is not shown because no reliable estimate of the hydrodynamic radius could be obtained due to uncontrolled swelling (R_H_(THF) > 325 nm, R_H_(THF)/R_H_(H_2_O) > 5)

Here, the semi-batch approach was used to synthesize three dispersions, labeled LL25-2, LL25-5, and LL25-8, with a fixed PEG5000/HFBMA molar ratio of 0.025 but with different amounts of EGDMA (molar EGDMA/HFBMA ratios of 0.02, 0.05, and 0.08, respectively) in order to crosslink the particles. The entries in Table 2 show that the particle size increased somewhat with increasing amount of the crosslinking agent. To examine whether the crosslinking was successful the particles were transferred to THF because the fluorinated monomer is soluble in THF and PEG exhibits partial solubility in THF [43]. All particles swelled in THF as compared to water as shown in Fig. 6. However, an increase in the EGDMA/HFBMA molar ratio led to a decrease in the relative increase of the hydrodynamic radius. Batch LL25, on the other hand, which was synthesized without a crosslinker, showed large, uncontrolled swelling on transfer into THF. Already at 2 % (mol/mol) crosslinker the swelling was reduced to within ∼10% of the value reached with the highest ratio of added crosslinker.

### PEG-grafted polystyrene particles

There are numerous studies of grafting PEG using macro-monomers in batch emulsion polymerization to give PEG-grafted polystyrene particles [36, 44–50]. Here, in a final application of the semi-batch emulsion polymerization method with slow initiator addition, it was used to make such aqueous dispersions of polystyrene particles grafted with PEG2000, labeled as batch PSSB in Tables 1 and 2. In addition, the corresponding batch polymerization, i.e., with all the initiator present at the beginning of the polymerization, as modeled after Brindley et al. [36], was used to generate a batch labeled PSB. Table 2 shows that the same particle size was obtained in both polymerizations, but the yield is poorer with the semi-batch method compared to the conventional emulsion polymerization. This shortcoming needs to be addressed before the synthesis protocol is viable for use at larger scale. In I, increase of the stirring rate led to improved yields. The initiator feed rate, which was not systematically investigated here, is likely another variable suitable for optimization aimed at improving yields further.

**Fig. 7:**
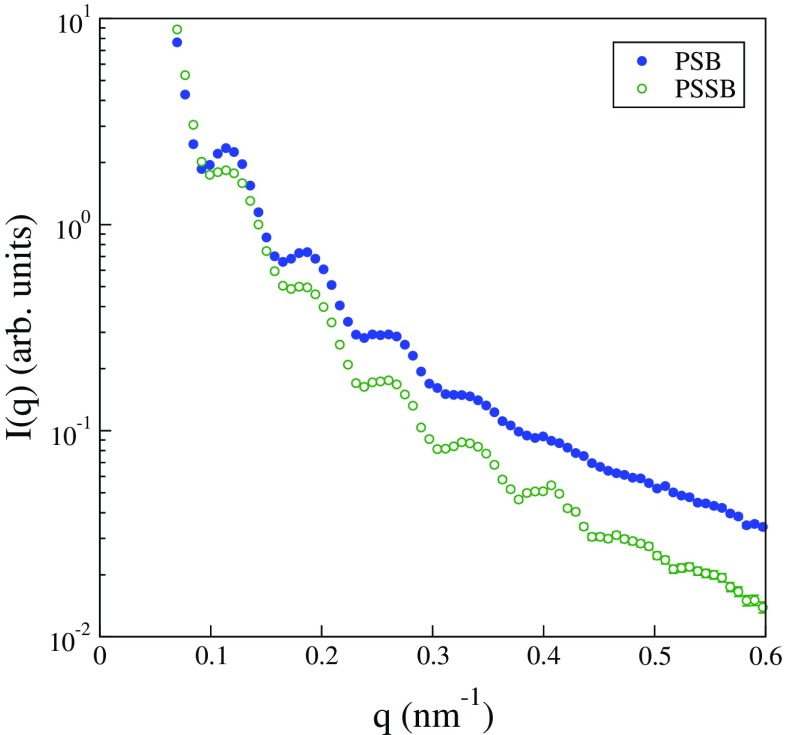
Scattering intensity recorded as a function of *q* and a 2-pinhole configuration for dilute dispersions of PEG-grafted polystyrene particles, as labeled

Due to the small density difference between particles and solvent, neither of these two dispersions was examined by DCP to determine the size distribution. Instead, SAXS measurements were made with the results shown in Fig. 7. Both dispersions result in form factors with several resolved minima. However, there are two differences worth noting. The first two minima of the semi-batch-produced dispersion, PSSB, are more smeared than those in the scattering from the batch-produced dispersion, PSB. At higher *q*, the PSSB batch exhibits several more resolved minima. The former effect suggests that the PSSB dispersion is characterized by a somewhat skewed size distribution in comparison with the PSB dispersion, which might be avoided by using a slightly lower molar ratio in the synthesis. The latter effect shows that the polydispersity is reduced with the semi-batch method for these PEG-grafted polystyrene particles, because the number of resolved minima or oscillations of the form factor is a sensitive function of the size polydispersity [51]. Both dispersions proved highly stable against Na_2_CO_3_ addition.

## Conclusions

The aim of this work was to demonstrate that the semi-batch emulsion polymerization method, previously used to make PEG-grafted fluorinated particles using a single PEG chain length [31], is flexible and can be used to graft both short and longer PEG chains on particles. For PEG macromonomers of lower molecular weight, some optimization is required if low polydispersities are needed because post-synthesis analysis has shown that smaller size particles build up as the molar PEG-macromonomer/HFBMA monomer ratio is increased. For the longer PEG5000 grafts, the molar ratio does not have the same effect on the size distribution, but some optimization might still be required because the particle size is strongly affected by the molar ratio. The particle size was also found to be governed by the overall concentration of fluorinated monomer. The particles that were synthesized with a crosslinking agent exhibited some limited swelling in THF, whereas non-crosslinked particles swelled uncontrollably. The degree of swelling could be controlled through the amount of added crosslinker. While yields appeared to be somewhat lowered by adopting the semi-batch method with slow feeding of the initiator, as observed, e.g., in a direct comparison with a standard batch polymerization involving styrene monomer, narrow size distributions were consistently generated provided the molar PEG-macromonomer/HFBMA monomer ratio was kept sufficiently low.
